# Experiences and lessons from structural interventions against COVID-19 in Addis Ababa, Ethiopia

**DOI:** 10.3389/fsoc.2024.1305549

**Published:** 2024-01-26

**Authors:** Kibur Engdawork, Ezana Amdework, Samuel Assefa, Desta Ayode, Getnet Tadele

**Affiliations:** Department of Sociology, College of Social Sciences, Addis Ababa University, Addis Ababa, Ethiopia

**Keywords:** COVID-19, structural intervention, lesson for future pandemics, challenges of interventions, low- and middle-income setting, Ethiopia

## Abstract

**Introduction:**

Fighting pandemics like COVID-19 requires implementing successful structural and behavioral interventions that attempt to change the social and political environments to increase adherence to preventive behavior among community members. However, studying structural interventions implemented during pandemics and their challenges remains to be uncharted territory in developing implemented countries.

**Objectives:**

Given this, we documented the experiences of implementing such interventions in Ethiopia with the aim of drawing lessons for future efforts to fight similar outbreaks in resource limited and low-income settings.

**Methods:**

We conducted a qualitative study between September and October 2021. Data were collected through face to face and telephone interviews from purposefully selected stakeholders from government and private sectors engaged in social interventions to prevent COVID-19. The systematization and the analysis of the data were conducted with MAXQDA 2020 software.

**Results:**

Ethiopia implemented structural and social interventions to respond to the COVID-19 pandemic. This included: developing national policy and guidelines, mainstreaming COVID-19 interventions to local organizations, implementing capacity development programs, and developing strategies to engage the community, through traditional institutions, in intervention activities. In addition, a mass communication approach was used to deliver risk messages. This yielded a promising result in slowing down the spread of COVID-19 in the capital of Ethiopia-Addis Ababa. On the other hand, competing interests, misconceptions, capacity constraints among professionals and organizations, limited capacity to enforce legislation and lack of motivation for change from the community side affected the implementation and the outcomes of interventions.

**Conclusion:**

Going forward, these challenges need to be taken into consideration when designing and implementing structural interventions to contain disease outbreaks effectively. The study highlighted that attempts to withstand pandemic in low- and middle-income settings shall successfully utilize local resources, act swiftly when pandemics outbreak and adjust themselves to the dynamic challenges and limitations of structural interventions.

## Introduction

On December 31, 2019, the outbreak of a coronavirus disease was reported from Chinese authorities in Wuhan City and named by the world health organization (WHO) as COVID-19 on January 30, 2020 and subsequently declared as public health emergency realizing the spread of the virus in other parts of the world outside of China (Güner et al., 2020). The COVID-19 pandemic has led to a dramatic loss of human life worldwide and presented an unprecedented challenge to public health (Mishra et al., 2020; WHO, 2021). The economic and social disruption caused by the pandemic was devastating. The impact was more overwhelming to low-and middle-income countries (LMICs) than global north due to dire limitations of healthcare infrastructure and expertise. According to the latest update report by WHO, as of 12 March 2023, there were over 760 million confirmed cases and over 6.8 million deaths globally (WHO, 2024). Although the vast majority of those infected survive, some survivors of COVID-19 are known to be at-risk for a variety of sequelae- a condition that has been known as post-acute COVID-19, commonly referred to as long COVID (Sudre et al., 2021; Ziauddeen et al., 2022).

Despite the number of new cases and death tolls declining overtime, the pandemic has continued to be a public health threat. Recently, WHO has lifted the Public Health emergency status of COVID-19 with a warning to the world that the disease should be managed alongside other infectious diseases (Laine and Moyer, 2023). According to WHO, worldwide, nearly 4.1 million new cases and 28,000 deaths were reported from 13 February to 12 March 2023, a decrease of 40% and 57%, respectively, compared to the previous month (WHO, 2024) of which Africa accounts for 12,712 (1%) new confirmed cases and 26 deaths (WHO, 2024). In Ethiopia, during the same period, 113 new cases were reported. A total of 500,163 COVID-19 confirmed cases have been detected and 7,572 deaths was reported as of 12 March 2023 (Ethiopia Public Health Institute, 2023). However, the true number of incident cases is likely to be underestimated due to a decline in testing nationally. In the same period, a total of 44,285,561 people have been vaccinated with at least one dose of vaccine, whereas about 37,796,736 people received full doses, bringing overall one dose and full dose coverage of 66.3% and 56.6% from the target population respectively (Ethiopia Public Health Institute, 2023).

The burden of COVID-19 has been well observed in recent studies. As an evolving disease, COVID-19 is associated with anxiety and depression (Pierce et al., 2020; Alat et al., 2023). The effect of COVID-19, however, goes far beyond this and is associated with social and economic stresses that disturb normal routine activities and interpersonal interaction. Fear of losing jobs, safety seeking behavior, avoidance of public spaces have been reported as the consequence of the pandemic (Arora et al., 2020).

Various Non-Pharmaceutical Health Interventions (NPHIs) have been used by different countries to control the spread of the virus, such as containment strategies, personal protective measures, economic support, and travel-related measures (WHO, 2020; Li et al., 2021). Evidence indicated that in a situation where non-pharmaceutical interventions are the major preventive options, public health social measures are paramount for outbreak management during the early phase of the pandemic (Hailemariam et al., 2021). According to WHO, public health social measures (PHSM) include non-pharmaceutical individual and societal interventions to control COVID-19 (Perra, 2021). Individual level interventions have been shown to yield promising results in preventing mental health problems associated with the pandemics (Alat et al., 2023). Social and behavioral prevention activities are considered the best strategies to reduce the healthcare burden, as they help slow transmission of the virus in the general population (Hailemariam et al., 2021). Experiences from Ebola outbreaks in Africa and the four decades of interventions on HIV/AIDS provide credible evidence about the effectiveness of public health interventions where resources for biomedical treatments were limited and interventions relied mainly on social and behavioral change models (Eaton and Kalichman, 2020). These experiences informed the need to create an enabling environment to support behavior change (Raguin and Girard, 2018). Enabling safer behaviors means addressing structures that constrain or enable people’s choices. A study by Suar et al. (2023) further showed that effective leadership can succeed in designing and implementing multisector context tailored interventions to reduce economic hardship and discrimination. The COVID-19 pandemic also requires a similar intervention approach driven by a social-ecological framework that acknowledges personal motivations, meaningful involvement of communities, and consideration of cultural and political contexts for building social capital, trust, and community cohesion to foster change (Jang, 2022). Evidence however suggests that the effectiveness of the public health and social measures in controlling COVID-19 depends on the intensity of transmission and must be continuously adjusted to the magnitude of transmission and capacity of the health system in a country with full participation and engagement of the community (WHO, 2020).

Lessons from the earlier pandemics also recommend the importance of preparedness measures essential to design, characterize, and evaluate interventions that can shape behavior (Calnan et al., 2018). A good practice documented in three West African countries (Guinea, Liberia and Sierra Leone) reported that in these countries, most of the selected readiness measures were instituted before confirmation of the first COVID-19 case, and response measures were initiated rapidly after the outbreak confirmation (Impouma et al., 2021). This suggests that the rapid readiness and response measures instituted by the three countries can be attributed to the lessons learned from past pandemics.

In response to the COVID-19 outbreak, the Ethiopian Government has taken prompt action during the initial phase of the pandemic, emphasizing containment measures and implementing a wide range of structural interventions to change the social, political and social environment to promote health behaviors. The government attempted to put in place robust surveillance systems, and rigorous isolation and quarantine operations. Additionally, steps such as the temporary closure of schools, limitations on public transportation, prohibitions on large gatherings, and the suspension of sporting and religious events have been taken to mitigate the spread of the virus (Baye, 2020). A state of emergency has been put in effect urging people to stay and work from home. The Ethiopian Ministry of Health and Public Health Institute also rolled out a national COVID-19 prevention and treatment guideline based on a recommendation by WHO. The guideline aims to contain the pandemic by guiding policy makers and health professionals at various levels (FMoH, 2020).

The capital of Ethiopia, Addis Ababa was the epicenter of the pandemic experiencing a sharp increase in the number of cases and death rate (Bizuneh et al., 2022). Although the administration exerted its effort to control the spread of the disease, available evidence showed that public adherence was a daunting challenge to effectively adopt recommended measures. A study conducted to assess public knowledge, attitude, and practice (KAP) during the first year of the pandemic in Addis Ababa found a moderate positive correlation between knowledge and attitude, whereas the correlations between knowledge and practice and attitude and practice were weak (Desalegn et al., 2021). A recent study on vaccine acceptance also showed that a considerable proportion of the people in Addis Ababa had concerns on COVID-19 vaccines and are unwilling to accept them (Dereje et al., 2022). This was due to misconceptions, negative attitudes, and use of social media as their primary source of information. Another study in selected towns including Addis Ababa reported that a considerable number of informants believed they were not at risk of contracting the disease (Harris et al., 2020). According to the qualitative findings of the same study, the perceived low prevalence of COVID-19 and the low perceived susceptibility to the disease seem to have contributed to a decline in the practice of preventive measures (Harris et al., 2020). Evidence also suggests intervention efforts in the country in general and in Addis Ababa in particular, faced myriads of challenges mainly related to public attitude and perception of risk, lifestyles, poor health system, limited resources and insufficient protective supplies (Okonji et al., 2021). A recent study showed that the weak testing infrastructure and statistical capacities in the country may mean that the full extent of the overall COVID-19 impact has been underestimated (Obande et al., 2021; Okonji et al., 2021).

Current evidence shows that the pandemic and its impacts have declined over time and WHO has declared that COVID is no longer an international public health emergency concern (Laine and Moyer, 2023). However, the experience has left the world a wake-up call on preparedness to tackle similar outbreaks in the future and raised both international and national interest to draw lessons from interventions. This study aims to investigate the effectiveness of structural interventions during the earlier period of the pandemic in promoting adoption of preventive actions, challenges encountered during implementation and draw lessons for future pandemic responses in low- and middle-income settings.

## Materials and methods

### Study design and setting

A cross-sectional qualitative study was employed, using key informant interviews conducted from September 05 through October 10, 2021. The study was conducted in Addis Ababa. Being the largest city in Ethiopia, Addis Ababa had a population of 4.8 million in 2020 (World Population Review, 2020). Addis Ababa was the most affected city compared to other cities in the country, and as such, was one of the epicenters of COVID-19 during the study period (Bizuneh et al., 2022).

### Participants

Study participants include various stakeholders from the government and private sectors engaged in social interventions to prevent COVID-19 in Addis Ababa. A purposive sampling technique was employed to recruit respondents. The inclusion criteria were institutions implementing public health social interventions in Addis Ababa and willingness to participate in the study. The researchers identified categories of institutional sectors such as government sector offices, NGOs and civic societies, law enforcement bodies, media authorities, religious institutions, and policymakers in the Addis Ababa Administration. We excluded two key informants who were not willing to take part in the intervention and who just joined the selected institution as they may have little information about implemented intervention. From each category, participants were selected with convenience sampling considering gender composition, stakeholder representation, accessibility, and willingness of the participants. Data collection progressed until saturation was reached as demonstrated by redundancy of information. Accordingly, 21 key informants participated in the study.

### Instruments

Guided by the social-ecological model and the literature, we developed an interview checklist. The interview checklist contained five with a total of 20 topical guides which explored the opinion of the key informants. These include: the extant social and structural interventions to address COVID-19, opinion about the effectiveness and feasibility of these interventions, major barriers influencing the interventions, and lessons for future recommendations. The set of issues in the checklist was revised in the course of the interview sessions to accommodate emerging issues.

### Data collection

Interviews were conducted by three trained and experienced male acadamics who had master’s and a PhD degree in social sciences. We submitted a letter of introduction to each organization. The organizations referred to their staff who were in charge of overseeing programs and interventions against COVID-19. We approached the staff and purposefully selected them considering our inclusion and exclusion criteria. Most of the interviews were conducted in two steps. In the first step, introductory contact was made with the potential informants to introduce them to the study, obtain verbal and written consent, and make appointments for interview dates. In the second, the actual interviews were conducted either face-to-face or virtually (telephone or zoom interviews). All interviews were conducted in the Amharic language. Field notes were taken for all interviews, while audio records were captured from some informants who consented to be recorded. After the data collection, a debriefing session was conducted with data collectors to discuss the key issues that emerged in the interviews, and challenges encountered. The interviews were conducted privately in the offices of the key informants. The average duration of the interview was 48 min, ranging from 40 to 56 min.

### Data analysis

Before data analysis, all the audio-recorded interviews were transcribed verbatim by the interviewers and then translated into English. The translations and the transcripts were checked by the PI of the study for quality and accuracy. A sample of interviews were also checked by professionals for validity and consistency of the transcriptions. MAXQDA 2020 software was used for data analysis. We then performed structural coding. Structural Coding applies a content-based or conceptual phrase representing a topic of inquiry to a segment of data that relates to a specific research question used to frame the interview (Saldaña, 2009). Research team members agreed on the coding and themes. To ensure reliability, three coders were involved to organize the data based on themes from the interview guides and themes derived from the data. The coders resolved overlapping themes and inconsistencies through regular meetings and incorporated additional themes identified through their discussion. After the completion of each interview, we summarized the main points and read them out to our interviewees for confirmation. Most respondents confirmed the data while some of the respondents provided additional information about their personal experiences. We used verbatim quotations as evidence to substantiate our themes and arguments. We provided detailed accounts about the contexts in which the interventions and the programs against COVID-19 were implemented.

### Ethical considerations

Addis Ababa University granted permission to conduct research. The study had minimal physical and psychological impact on participants. Participation in the study was voluntary. Confidentiality was maintained during the data collection, analysis and report. Personal data are not mentioned in this report. Written informed consent was obtained from all participants.

## Results

Most informants claimed the target of their interventions was to achieve optimal preventive measures among the populace in Addis Ababa. As one informant from the National COVID-19 Taskforce noted, “our primary objective was to attain the COVID-19 prevention measures as outlined in the WHO and Ethiopian Public Health Institute (EPHI) guidelines.” The optimal preventive measures most stakeholders envisioned include improving face mask use, hand washing, and physical distancing. Some organizations also included self-isolation, adherence to testing, vaccine and treatment as primary expected outcomes of their behavioral interventions. The study identified an arsenal of interventions that were implemented by various stakeholders in the city to stimulate and enforce the adoption of these preventive measures, broadly categorized into structural interventions and risk communication.

### Structural interventions

#### Policy/legal frameworks

A State of Emergency was declared on April 8, 2020. Following this national decision, several proclamations and directives were issued that introduced measures such as a nationwide ban on public gatherings, making the wearing of masks compulsory outdoors, and regulating the operation of transportation services, hotels, and restaurants under reduced capacity, etc. According to participants, these measures were widely practiced in Addis Ababa during the initial phase of the pandemic. Most key informants also share the idea that these policy and legal decisions have significantly contributed to the slowing down of the spread of the coronavirus. A notable measure taken by the Addis Ababa city administration was the formation of a COVID-19 task force spearheaded by the city council and comprising various sectors. The task force was the central command responsible for developing a city-wide COVID mitigation plan and guiding and monitoring its implementation. Similar interim structures were formed at lower levels to facilitate intersectoral collaborations. As a key informant noted in this regard, “*the regional COVID-19 intervention policy has promoted stakeholders’ collaboration established across all sectors during the outbreak of the pandemic*.”

#### Mainstreaming COVID interventions

The institutional service delivery system is another locus of structural interventions that received a momentum in COVID-19 prevention. At the institutional level, most organizations have undertaken a covid adaptation strategy and planned to engage in COVID-19 prevention interventions by setting up interim structures and allocating resources to deal with the pandemic. Internal work guidelines were developed tailoring the national COVID-19 prevention guideline such as work from home where possible, arrangements for a shift work system in offices and schools, application of covid preventive measures in service delivery settings, etc. An informant from the NGO sector noted, “*Our COVID-19 adaptation strategy enabled all programs to mainstream COVID-19 prevention in their programmatic operations*.” Informants also noted that many organizations made funding arrangements or were soliciting funds to supplement the prevention activities. A key informant from an NGO stated, “*the senior management requested our donors to shift some of the budget from the WASH project to COVID-19 prevention program.”*

#### Capacity building

Informants highlighted that there were clear capacity gaps in surveillance and testing, contact tracing, risk communication, etc. at the initial period of the pandemic. Accordingly, different measures were taken by the city Administration in line with the national measures to bridge this gap through capacity building training, financial support from stakeholders, facilitating collaborations, and partnership with various actors. The role played by professional associations such as Ethiopian Public Health Associations (EPHA) was noted as being highly instrumental. The following excerpts provide insights into efforts undertaken in capacity building. “*We were able to produce 4,500 well-trained health workers in Addis Ababa and at national level. This contribution created significant backup to the regional and national interventions efforts*.” Due to the proactive measures taken by the city administration task force, progress was made in the testing capacity with adequate technical and financial support rendered to this effort. As a key informant from one sub city office mentioned, unlike during the outbreak period, all the health Centers in the city are now well equipped and are able to provide COVID-19 testing services.

#### Community engagement

Most of the key informants have reiterated the use of community-based structures such as mobilizing community organizations (e.g., self-help associations) influential role models, youth volunteers, community policing structures, etc. for disseminating messages in the community, reaching out vulnerable populations, and delivering support to those affected by the pandemic. “*During the outbreak of the pandemic community-based organizations. NGOs and religious leaders mobilized residents to support poor families*.” A key informant from Addis Ababa Emergency Operation Center (EOC) describing the role of community participation in promoting preventive actions reported the following:

The risk communication team in our office is responsible for mobilizing the community on prevention activities. A family health team also works with the community structures moving door to door with youth volunteers to sensitize the community about COVID-19 and its preventive measures. They also report affected individuals to the case management team for immediate actions.

However, some informants argued that the community engagement process is not as inclusive as it should be. It appears that in most cases the process sticks to the top-down model of operations. Community groups and leaders have taken the role in implementing the directives but played little role in planning, codesigning contextually feasible programs, and in monitoring and evaluations. Explaining this, a key informant mentioned, “*it should not be limited to providing awareness by community leaders. Efforts should be made to involve the community to plan together in a bottom-up fashion of operation and participate in decision-making process as well*.”

### Challenges influencing structural interventions

#### Competing interests

One of the main barriers mentioned by the informants affecting the adoption of preventive behavior among individuals was the influence of other competing interests preoccupying their attention. It was broadly noted that during COVID-19 partial lockdown period, families with low economic status, particularly women, experienced increased financial hardship, food insecurity, domestic violence, and mental health challenges. Due to this, people leading subsistence life were left in greater dilemma to deal with adherence to preventive measures and striving for economic survival. A key informant from the interreligious council reported, “*many poor people were out of their homes searching for opportunities to earn daily income for their survival giving a deaf ear to restrictions of movements, staying at home and social distancing*.” Other informants also explained the impacts of competing interests in terms of significant incidents such as national elections, conflicts in the country, etc. that may have forced reallocation of resources to these events during the pandemic. A key informant form Bole Sub city health office noted that most people were overwhelmed with news about the conflict in the northern part of Ethiopia and preoccupied with the then political instability than the COVID-19 messages.

#### Misconceptions

Most informants pointed out the prevailing misconceptions and negligence among many people in the city as a hindering factor for taking sustained preventive measures. Misconceptions included individuals denying the presence of the Coronavirus or misunderstanding the risk communication messages. One key informant said, “*during the outbreak, people considered COVID-19 as a false fabrication, not a disease.*” Misconceptions were also reinforced by the infodemic during the pandemic where most people were indiscriminately taking up information distributed from noncredible sources such as social media. As one key informant from the media sector reported, “*many people considered the pandemic as a doom’s day evil with no solution, and many others also believed vaccines to be microchips maliciously distributed by western countries. This created vaccine hesitancy and skepticism towards other preventive measures.*” Some informants also claimed that such misconceptions and negligent behaviors against COVID-19 stem from the peoples’ low perceived risk or low perceived susceptibility to the coronavirus. The following excerpt vividly describes this assertion:

There are people who do not use facemasks and we also see less adherence to physical distancing measures, especially among young people assuming they had low risk or vulnerability to the virus. During our campaign, we used to receive feedback from some young people telling us that coronavirus would not affect them as they are regularly doing physical exercises and eat well.

#### Inaccessibility of personal protective equipment

Another impeding factor underlined by informants for the implementation of measures for prevention of COVID 19 was the shortage of personal protective equipment (PPE) such as facemasks and scarcity of hand cleaning solutions such as lack of water, soap, alcohol, and sanitizer. A key informant from one of the City’s schools noted “*we often inform students to adhere to hand hygiene practice, however, shortage of water and soap in our school and in students’ homes were a critical problem discouraging our students and staff to consistently adopt this behavior*.” Shortage of water was a critical barrier reported in most sectors that influenced consistent practice of hand hygiene, especially in communities with limited resources. Emphasizing on this problem, a key informant noted, “*as a good practice, hand hygiene and washing were a regular routine early in the pandemic, and locally made hand washing stations were placed outdoors in several institutions and public places, but soon these became non-functional due to lack of water*.” Inability to afford and inconsistent access to these protective materials have created an intermittent adherence to preventive measures. In connection to this issue, a journalist reported, “*in an interview we made regarding the practice of facemask use, I got the impression that many people are in favor of using masks but are not doing so, claiming that it is very expensive*.”

#### Capacity constraints

Key informants also identified various capacity limitations affecting their efforts to promote prevention activities. The main challenges reported in this regard were shortage of financial resources and the lack of trained and skilled human resources. Health professionals and informants from academic institutions highlighted the shortage of trained health communication professionals as the main challenge in health promotion activities at the national and city level. They suggested the need to provide more attention by the higher education sectors and public health institutions to produce skilled human resources in this aspect. Explaining this point, a key informant from a professional association reported, “*Social and behavioral change communication (SBCC) is the best tool for public health emergencies…higher institutions should expand such disciplines or academic fields to produce more workforce in this profession*.” Those who reported financial resource limitations stated that the challenge led them to carry out irregular intervention and forced them to function under capacity. “*Our financial capacity did not allow us to work on prevention and awareness creation activities as much as we would have liked*.” (Key informant from Addis Ababa Bureau of Women and Social Affairs).

#### Limited law enforcement

Despite essential legal frameworks being set to augment COVID-19 prevention endeavors, inconsistent law enforcement practices observed in the city during the pandemic were reported as one barrier to COVID-19 intervention. Most informants acknowledged better law enforcement activity was employed in the transport sector than others. “*The police were cracking down on controlling facemask use of passengers and carrying capacity of transport vehicles as per limits, but little effort was made on other service sectors*.” Others also reported their concerns over the reluctance of some law enforcement officers themselves who did not take protective measures while on duty. One informant said, “*If law enforcement officers do not abide by the law, their legal measures would create a double standard; leading others to follow suit*.”

#### Lack of motivation for change

Adoption of preventive actions was also challenged by the apathetic response of the public toward COVID-19 prevention measures due to longstanding and strong cultural and religious norms. There were instances where some people were influenced by their strong social norms and religious stands. As one of the key informants pointed out “*many people were disregarding public health measures; seeking solutions to come only from their faith*.” Another informant from the NGOs sectors also mentioned, “*the long-standing culture of practicing social events, compromised the public acceptance of preventive measures against the pandemic*.” This was reported as one of the hindering factors specially for social distancing and facemask use and vaccine acceptance.

### Risk communication interventions

Mass communication was the most widely employed strategy reported by informants in delivering risk communication messages to the residents of Addis Ababa. As a key informant from the NGOs sector noted, “*we used community-based campaigns, religious institutions, TV and radio programs, and reached more than 3 million people*.” Another informant from the government trade sector pointed out, “*we passed COVID-19 messages across to the business community and the public through our telegram channel and Facebook pages*.” Similarly, a key informant from the transport sector mentioned, “*… we conducted rounds of big public campaigns accompanied by marching bands, city buses, traffic police vehicles around public squares and distributed fliers informing facemask use in public transports*.”

Another set of reported interventions were group-based risk communication strategies which were delivered to smaller groups of target populations in the community or institutional settings. Informants mainly from the health sector, NGOs, and community-based organizations highlighted a range of group interventions. These include community mobilizations, community conversations, trainers of training for a group of volunteers who would transfer knowledge to community members, workshops, and neighborhood group discussions on COVID-19 prevention measures. Explaining these interventions, a key informant from a city’s health office reported, “*in collaboration with NGOs we trained youth volunteers to disseminate information about risk factors and preventive messages in marketplaces, slum neighborhoods, and taxi stations, which made an enormous contribution to controlling the spread of the virus*.” Some key informants also pointed out social networks as one of their communication strategies employed to reach groups and individuals in the community. Through this approach, they were able to disseminate messages on preventive actions to a group of people in social circles. An informant from the NGO sectors noted, “*we have employed a community-based risk communication strategy through the network groups where trained members provided information about face covering, handwashing, and physical distancing to individuals in their social circle*.”

Various institutions also widely used local *iddirs* (self-help voluntary association that serves as economic and social insurance at times of death and other crises) and religious leaders to reach out to community groups and individuals with COVID messages. As one of the NGO leaders mentioned, “*we set up community-based information dissemination centers in each sub city. We worked with the association of iddirs and religious leaders to disseminate messages about the pandemic. This was effective because we were able to reach families with information on prevention measures at an early stage of the pandemic*.” Another prominent example of the individual and group-based risk communication approach applied was the rolling out of a family health team (each team comprising 5–8 people drawn from the health, education, and social sectors) by the Addis Ababa health bureau. This strategy was extended to all-sub cities and woreda-level structures that helped to successfully perform door-to-door COVID-19 surveillance, referral, and sensitization works, especially during the first wave of the pandemic.

### Risk communication messages

Most key informants hold the impression that risk communication messages disseminated were informative and met the EPHI guideline. However, when it comes to the *appropriateness* of the risk communication messages, informants reflected mixed findings. Some informants claimed a contextualized application of the preventive messages was used to make them suitable to their audiences and constituencies. For instance, a key informant from an NGO reported, “*developing preventive messages in* var*ious languages targeting children and their families via a TV spot, TV programs and through the religious institutions*.” Others also noted the effectiveness of risk communications claiming that the wider public misconceptions and bewilderments surfaced at the initial wave of the pandemic gradually dwindled due to extensive and diversified behavioral intervention messages communicated at all levels. Describing this point, a unit leader from Addis Ababa Emergency Operation Center (EOC), explained, “*initially messages were focusing mainly on the proper use of face ask, hand hygiene, and physical distancing. Alternative messages such as open ventilation and taking vaccinations were introduced to the public at the later stage to consolidate the prevention*.”

Some informants suggested the risk communication messages should consider the unintended positive benefits of the pandemic as motivators to reinforce positive behavior change (improved facemask use, hand hygiene, and less physical contact) to prevent from other infectious or contagious diseases. Informants from the health sector claimed that communicable diseases and respiratory infections have substantially declined since the outbreak of the pandemic due to behavioral and structural intervention such as facemask and hand sanitation becoming a new normal. The key lesson to draw from this experience is that effective communication messages should involve content to strengthen people’s perception about the potential benefits of protective measures.

However, many informants argued that some communication messages lacked clarity and were disseminated without paying sound attention to the target audience and the context they represent. As one informant from Ethiopian Public Health Professionals Association suggested, “*messages were mostly developed with a cut and paste approach without being contextualized to the objective realities on the ground. Messages should be evaluated with an interdisciplinary team of experts paying attention to the context*.” Describing this view, another key informant also highlighted, “*… for example, some short messages such as ‘When you are sick stay home’ was highly confusing and not clear specially for people with low literacy unless supported with further clarifications by skilled health professionals*.” An expert at the Addis Ababa EOC noting the difference between the concept of physical and social distancing reported, “*this should be clarified properly to the audience. These two terms have different meanings, but people were using them interchangeably. It does not mean avoiding socializing with people but keeping physical distance*.” There was also inconsistency in using some messages by various organizations such as in informing and enforcing physical distancing. On this point, a key informant from Ethiopian Inter-religious council explains “*I saw posted messages in many places in the city to inform the audience to maintain at least 2 meters physical distance while the EPHI’s guide required one meter. These messages should be carefully evaluated and presented to the audience*.”

Participants also reported gaps in the message development process. For instance, an official from the Addis Ababa City Health Bureau highlighted, “*the message development did not undergo a rigorous assessment of the context of the community or the target audience. This was partly due to emergency response required during the outbreak of the pandemic and limited resources or capacity*.” The other main challenge reported regarding the appropriateness of message was the lack of pretesting. Explaining this point, an expert from the Ethiopian Orthodox Church noted “our office employed a communication expert on a contract base for designing messages and messages were designed considering various target groups, but we did not conduct pretesting the drafts before distributing them to the public.”

## Discussion

The present study illuminated several social interventions in Addis Ababa, Ethiopia that could contribute to adopting optimal preventive measures against public health emergencies like COVID-19. The coronavirus pandemic is not a concern of the health sector only. Hence, a key takeaway of our qualitative work is that the diverse impacts of the pandemic on social, economic, and psychological life of the people in Addis Ababa required multipronged interventions to address the challenges holistically. The complex nature of the pandemic and its variability also demand employing a combination of preventive measures in tandem to achieve optimal results in preventive practices. Indeed, this will be made possible by revitalizing and strengthening intersectoral collaborations among stakeholders which is instrumental to improve preventive behaviors, and ultimately to halt transmission of COVID-19 and evolving new variants. In our findings, heightened collaborative effects and a swift multisectoral engagement plan designed by the Addis Ababa City Administration and their concerted actions demonstrated at the initial phase of the pandemic have meaningfully contributed to slowing down the pandemic. This proactive synergy has left a lauder lesson informing all actors to remain steadfast on sustained collaborations to overcome the multifaceted challenges during such outbreaks. This finding is consistent with findings from countries hit by past disease outbreaks, suggesting that greater decreases in incidence and mortality of COVID-19 were shown when authorities enforced collaborative interventions at the early stages of the pandemic (Kraemer et al., 2020; Iezadi et al., 2021; Piovani et al., 2021).

Our findings highlighted the essential practices of community-based interventions as fundamental learning points in response to COVID-19. Participation of the community was significant in supporting high-risk/vulnerable people to cope with the economic and social challenges caused by the pandemic. Involving survivors and vulnerable groups in the prevention process is also instrumental. The active role played by religious institutions, youth volunteers associations, professional associations, and community policing structures, in disseminating COVID-19 messages to their constituencies, assisting in contact tracing and mobilizations of resources was a prominent example of this endeavor. Good practices seen under these community-based initiatives inform the need to enhance the capacity of community-based organizations and empower local institutions as part of the preparedness plan of the regional administration. The fundamental benefit of community engagement in the wake of the pandemic has been documented in many research findings (Anoko et al., 2020; Smith and Judd, 2020). Despite these initiatives, our finding suggested the need to make community engagement more meaningful in the sense that participation should be scaled up from informing or consulting to meaningfully involving them to be able to jointly design and implement and monitor interventions. Looking along the spectrum of participation, we found community’s initiative to own interventions and to facilitate public adherence to preventive behavior to be essential. Experience gained from the Ebola response also asserts that implementing community-led action to control COVID-19 are likely to be successful (Smith and Judd, 2020).

Findings pointed to the need for relevant and appropriate messages. One of the key limitations in the message mapping process during the initial phase of the pandemic was the fact that messages were not informed by a community assessment and little effort was made to identify context-specific concerns of various stakeholders and the needs of diverse vulnerable populations. Communication messages should be tailored to the context and targeted to the appropriate groups. This should be made possible through context assessments and participation of the community. Behavior change is more likely if the target population is given the platform to actively participate in the intervention process. This lesson goes in line with the experience of risk communication in times of disasters that suggests information should be adapted to the literacy needs of the people it intends to reach, with special attention for those who are the most vulnerable in pandemics (Baral et al., 2013; Corbin et al., 2021).

Our findings also provided implications for message framing in risk communication programs. Risk communication interventions discussed by the participants indicated that messages disseminated to the public need an updated review. Periodic assessment of the effectiveness of the COVID-19 messages is crucial to take an up-to-date response that fits to the realities on the ground. For instance, people’s reaction and response to COVID-19 during the first phase of the pandemic was full of fear, panic, stigma, and confusion, but the perceptions of threat changed over time. Hence, the tone of the message and its contents should change along with the evolving realities of the pandemic. Warning messages during the initial periods of the pandemic might have increased the adoption of preventive practices. However, similar messages may not work later during the pandemic as the fear among the residents has gradually subsided. This is in line with the social-ecological model that suggests the stage of the epidemic will determine the risk of disease acquisition for the individual (Baral et al., 2013). Therefore, risk behavior and the adoption of preventive action should be interpreted within the context of the stage of the epidemic.

There were also unintended outcomes of interventions. For example, the unintended positive impacts produced from social interventions (such as face covering, physical distancing and hand hygiene) in reducing other communicable and contagious diseases would motivate the public to positively receive messages on preventive practices. This is in line with the principle of various behavioral change theories such as Health Belief Model (HBM) which holds that, when perceptions of benefits are substantially greater than costs, people are more likely to intend to search for health information and enact health-protective behaviors (Karimy et al., 2021). Similarly, some studies on facemask use also identified that perceived benefits of mask use did have significant effects on mask-wearing compliance as well (Tadesse et al., 2020; Yasa et al., 2021). Hence, this finding points to the need for including all potential benefits of protective measures in risk communication approaches for COVID-19, to maximize behavior change outcomes.

Our findings acknowledged the importance of water, sanitation, and hygiene (WASH) in the response to COVID-19. During the pandemic, various initiatives that relate to the intensification of behavior change and awareness-raising campaigns for the promotion of handwashing measures were widespread in the city and have been widely adopted by the residents. Installation and operation of hand washing stations in public spaces was a common phenomenon during the pandemic. However, the extant huge disparities in access to WASH facilities posed a serious challenge. Therefore, a prominent lesson one can draw from our findings is the need to build resilience against future outbreaks through the integration and expansion of WASH intervention programs and promoting behavioral changes in environmental sanitation and personal hygiene. Properly managed WASH services are needed to support at-risk populations to build resilience against future pandemics. Specially, improving access to water supply and sanitation in slums is an urgent issue for building a resilient city not only to address COVID-19 but also for other future infectious diseases.

Our study provided better insights drawn from multi sectoral responses against COVID-19 in low- and middle-income settings. Data were collected from a wide range of key informants that represent diverse perspectives. Despite the richness of qualitative data, the study also has some limitations. As our findings are based on qualitative data only, further investigation may be needed to validate the findings with quantitative data to ensure generalization into other settings. Moreover, while our participants were assured of confidentiality and their responses appeared to be very candid, we cannot rule out the possibility of socially desirable responses. Gathering information only from key informants through in-depth interviews might affect the transferability of the result.

## Conclusion

What has been learnt effectively from the current COVID social interventions shall have broad relevance for managing future public health emergencies. A louder message echoed from the past 3 years trajectory of the pandemic is the need for setting a robust public health emergency preparedness strategy that effectively challenges future emergencies of similar kind. The key lessons from this study are manifold. Behavioral change interventions should be context specific and sensitive to the dynamism of the evolving COVID-19 trajectory. The current trend of risk perception and adaptation of the public to live with the pandemic could inform the need to modify risk communication strategies focusing more on the potential benefits of preventive actions. Consistent and sustainable coordinated efforts among stakeholders are necessary to contain COVID-19 and future pandemics through context-specific prevention strategies. Community based responses are vital to effectively control the social transmission pathways, which may be particularly important to reach marginalized populations. Hence, extensively employing existing community engagement structures is paramount to maximize adoption of preventive actions against pandamics. Since dynamics of pandemics can change, interventions targeted to adopt preventive actions need to follow dynamic trends.

## Data availability statement

The raw data supporting the conclusions of this article will be made available by the authors, without undue reservation.

## Ethics statement

The studies involving humans were approved by Addis Ababa University, Department of Sociology. The studies were conducted in accordance with the local legislation and institutional requirements. The participants provided their written informed consent to participate in this study. Written informed consent was obtained from the individual(s) for the publication of any potentially identifiable images or data included in this article.

## Author contributions

KE: Conceptualization, Data curation, Formal analysis, Funding acquisition, Investigation, Methodology, Project administration, Software, Supervision, Writing – original draft, Writing – review & editing. EA: Data curation, Formal analysis, Funding acquisition, Investigation, Methodology, Software, Validation, Writing – original draft, Writing – review & editing. SA: Validation, Writing – review & editing. DA: Data curation, Formal analysis, Investigation, Supervision, Writing – original draft. GT: Funding acquisition, Validation, Writing – review & editing.
